# MALS: an efficient strategy for multiple site-directed mutagenesis employing a combination of DNA amplification, ligation and suppression PCR

**DOI:** 10.1186/1472-6750-9-83

**Published:** 2009-09-24

**Authors:** Alexey A Fushan, Dennis T Drayna

**Affiliations:** 1 National Institute on Deafness and Other Communication Disorders, National Institutes of Health, Bethesda, MD 20892, USA

## Abstract

**Background:**

Multiple approaches for the site-directed mutagenesis (SDM) have been developed. However, only several of them are designed for simultaneous introduction of multiple nucleotide alterations, and these are time consuming. In addition, many of the existing multiple SDM methods have technical limitations associated with type and number of mutations that can be introduced, or are technically demanding and require special chemical reagents.

**Results:**

In this study we developed a quick and efficient strategy for introduction of multiple complex mutations in a target DNA without intermediate subcloning by using a combination of connecting SDM and suppression PCR. The procedure consists of sequential rounds, with each individual round including PCR amplification of target DNA with two non-overlapping pairs of oligonucleotides. The desired mutation is incorporated at the 5' end of one or both internal oligonucleotides. DNA fragments obtained during amplification are mixed and ligated. The resulting DNA mixture is amplified with external oligonucleotides that act as suppression adapters. Suppression PCR limits amplification to DNA molecules representing full length target DNA, while amplification of other types of molecules formed during ligation is suppressed. To create additional mutations, an aliquot of the ligation mixture is then used directly for the next round of mutagenesis employing internal oligonucleotides specific for another region of target DNA.

**Conclusion:**

A wide variety of complex multiple mutations can be generated in a short period of time. The procedure is rapid, highly efficient and does not require special chemical reagents. Thus, MALS represents a powerful alternative to the existing methods for multiple SDM.

## Background

The introduction of multiple mutations in the same gene is often necessary for a wide range of studies, including studying gene expression and protein structure and function. Thus, site-directed mutagenesis is a central method in molecular biology. A number of strategies have been developed to simplify the generation of multiple mutant sites within a target sequence [1,2].

A popular approach employs several pairs of mutagenic primers for sequential rounds of mutagenesis. This procedure is robust, however it is time-consuming because it requires a subcloning procedure between rounds of mutagenesis [3]. Another strategy utilizes combining mutagenic oligonucleotides in the same reaction [4-7]. Non-PCR based template amplification in combination with ligation of growing DNA strands reduces the rate of spontaneous errors. Despite the advantages, this procedure also has limitations, such as restrictions in the number, sizes and types of mutations that can be introduced simultaneously [2]. Alternatively, a few DNA fragments, each carrying mutations, can be sequentially connected to generate a joined product with multiple mutations [8]. Selection measures such as nested PCR or enzymatic digestion have been developed for removal of wild-type DNA [9-11]. However, the possible combinations of DNA molecules grow geometrically with the number of fragments generated, only one which is the desired product. The need for resolution of these products greatly reduces the overall efficiency of the method.

In this study we described a rapid and efficient PCR-based strategy for multiple SDM that has several advantages over existing approaches. The method allows the creation of a wide variety of mutations in any combination, including sequence insertions, deletions of different lengths, and complex nucleotide exchanges. The mutagenesis is highly efficient (60-100% in our model experiments). Multiple complex mutations in the same DNA template can be introduced without intermediate subcloning, thereby permitting the generation of sequences with multiple mutations in a short period of time.

## Results and discussion

**Figure 1:**
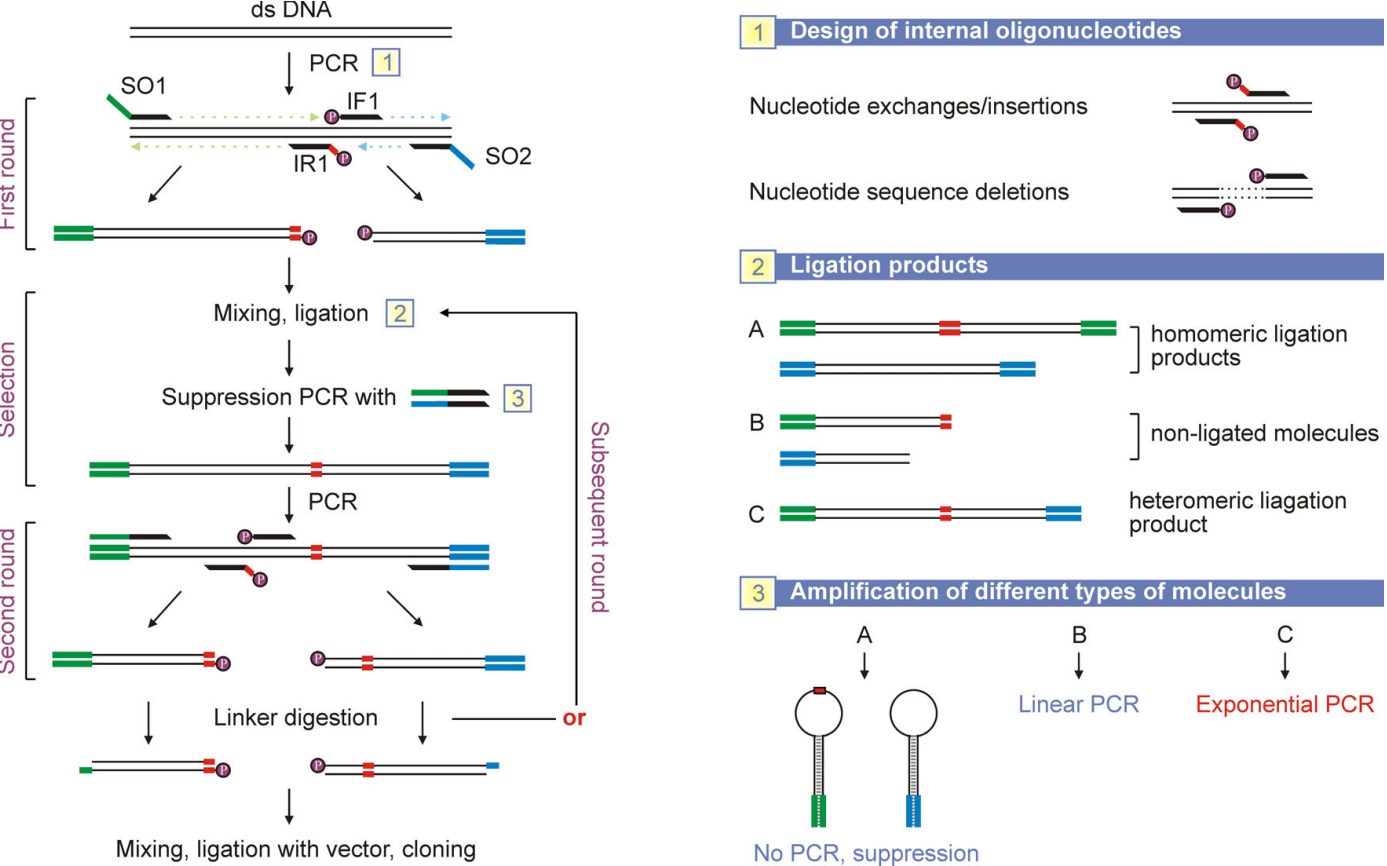
**The MALS mutagenesis procedure**. Target DNA is individually amplified with two pairs of oligonucleotides SO1/IR1 and SO2/IF1. IF1 and IR1 are phosphorylated (rose circles), while SO1 and SO2 are dephosphorylated. The desired mutation is incorporated at the 5' end of IR1 oligonucleotide (shown in red). Panel 1 on the right shows the types of mutations available with MALS. PCR-generated DNA fragments are ligated. The resulting DNA population consists of homomeric ligation products (type A), non-ligated DNA fragments (type B), and molecules representing full length target DNA (type C) (panel 2 on the right). The entire DNA population is then amplified with suppression oligonucleotides SO1 and SO2. Intramolecular hybridization of inverted repeat sequences prevents efficient replication of type A molecules, while type B molecules amplify linearly (panel 3 on the right). Only heteromeric ligation products (type C) amplify exponentially. The resulting DNA population predominantly consists of type C molecules. An aliquot of the PCR mixture is used for the next round of mutagenesis employing internal oligonucleotides specific for another region of target DNA. Finally, SO1 and SO2 suppression sequences (green and blue, respectively) are digested with restriction endonucleases and DNA fragments are ligated with linearized vector for subsequent subcloning.

**Figure 2:**
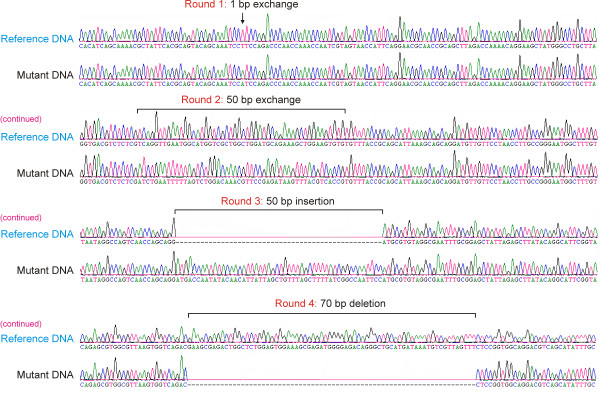
**Sequence traces of mutated and initial DNA templates demonstrating nucleotide alterations that were sequentially introduced during four rounds of MALS procedure**.

The principle scheme of the method is shown on Figure 1. MALS procedure (**M**utagenesis by **A**mplification, **L**igation and **S**uppression PCR) allows the generation of all types of mutations depending on the design of the internal oligonucleotides used (Figure 1). The maximal sizes of nucleotide exchanges and insertions are restricted only by the lengths of internal oligonucleotides, and there are no length restrictions for sequence deletions. To demonstrate the efficiency of the method we sequentially introduced four different nucleotide alterations in a model 4 kb DNA sequence originating from the genomic DNA of phage λ (Figure 2).

**Figure 3:**
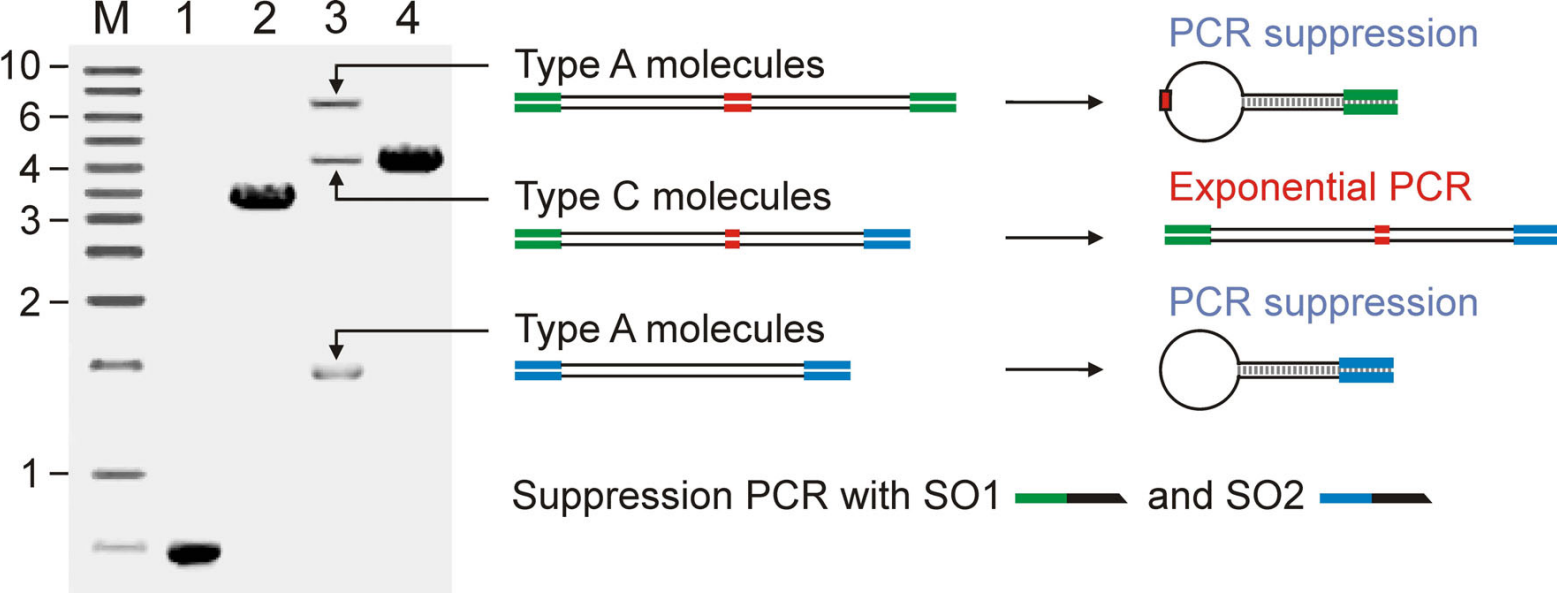
**Agarose gel electrophoregram demonstrating the types of molecules forming during one individual round of mutagenesis**. Lanes 1 and 2, PCR of target DNA with SO1/IR1 and SO2/IF1 oligonucleotides; Lane 3, homomeric (type A) and heteromeric (type C) DNA molecules formed during ligation of SO1/IR1 and SO2/IF1 DNA fragments (also, see Figure 1); Lane 4, suppression PCR with SO1 and SO2 oligonucleotides. The scheme on the right shows a graphical representation of type A and type C molecules that are present in lane 3 of the gel. During suppression PCR, intramolecular hybridization of inverted repeat sequences prevents binding of SO1 and SO2 oligonucleotides to the type A molecules that prevent their effective replication. Type C molecules amplify exponentially (see Additional File 1 for further details). Lane M, DNA size ladder with indicated positions of 1, 2, 3, 4, 6, and 10 kb DNA fragments (GeneRuler 1 kb DNA Ladder, Fermentas).

Initially, we introduced 1 bp nucleotide exchange in the target DNA. Double stranded DNA template was amplified with two non-overlapping pairs of oligonucleotides (Figure 3, lanes 1 and 2). External oligonucleotides SO1 and SO2 were dephosphorylated and consisted of template-specific sequences and high-melting suppression sequences having no similarity to each other (Table 1, Additional File 1). In addition, two different restriction recognition sites (absent from target DNA) were introduced into the external oligonucleotides for subsequent cloning. The desired mutation was incorporated at the 5' end of one of the internal phosphorylated oligonucleotides (Table 1, oligonucleotide IR1). PCR amplifications were performed in separate tubes to minimize generation of non-specific PCR products.

**Table 1:** Oligonucleotides used in this study.

**Name**	**Nucleotide sequence (5'-3')**	**Orientation**	**Mutation introduced**
Suppression oligonucleotides (*Nhe*I and *Hind*III restriction recognition sites are marked in bold)			
SO1	GTATGGACACTGCTGCGCGG**GCTAGC**TCCGGATGCGGAGTCTTATCC	Forward	-
SO2	CGCCCGTGACACTCTCCAGC**AAGCTT**CCATAGCAGCCATCACATCAGT	Reverse	-
Mutagenic internal oligonucleotides (mutagenic nucleotides are marked in bold)			
IF1	TCCAGACCCAACCAAACCAATCG	Forward	1 bp exchange
IR1	**T**GGATTTGCTGTACTGCGTGAATAGC	Reverse	
IF2	**GTTCCGAGATAAGTTTACGTCACCG**TGTTTACCGCAGCATTAAAGCAG	Forward	50 bp exchange
IR2	**GTTTGTCCAGACTAAAAATTCAGAT**CGAGAGACGTCACCTAAGCAGG	Reverse	
IF3	**TTAGCTTTTATCGGCCAATTCCATG**CGTGTAGGCGAATTTGCGGAG	Forward	50 bp insertion
IR3	**ACAGCTAATAATGTTGTATATTGGT**CATCCTGCTGGTTGACTGGCCTA	Reverse	
IF4	CTCCGGTGGCAGGACGTCAGCA	Forward	70 bp deletion
IR4	GTCTGACCACTTAACGCCACGC	Reverse	

If only one mutation is needed, the resulting PCR fragments can be digested with specific restriction endonucleases and directly ligated with vector for subsequent subcloning. To perform multiple-site mutagenesis, PCR fragments obtained during amplification are mixed together in equal molar proportion and ligated without prior digestion. Ligation of blunt-ended DNA fragments can be done at room temperature (20-25°C) in 10-30 min with high concentrated T4 DNA ligase (30,000 Weiss units/ml, #M0202 M, New England Biolabs).

A similar mutagenesis procedure which utilizes two separate PCRs to amplify two halves of a complete gene using four primers was developed earlier, and is known as a connecting SDM [1]. In the connecting SDM, two resulting PCR products are ligated to form fused DNA carrying mutation situated in the middle. However, PCR fragments become ligated in all possible combinations, and the final DNA mixture contains multiple DNA species. This does not allow a repeat mutagenesis procedure without prior subcloning for selection of the correct mutants. Connecting SDM was proposed as a single round mutagenesis method designed for the introduction of a single mutation with subsequent subcloning [1].

We applied a selective amplification based on the PCR suppression effect [12] for the separation of desired mutant molecules from other types of molecules formed during ligation and present in the entire DNA population. This procedure enables one to avoid time- and labor-intensive subcloning. Suppression PCR has been used successfully in a variety of applications: genome walking [12], DNA subtractive hybridization [13], and targeted genomic differential display [14]. In suppression PCR, an inverted repeat is incorporated in the ends of DNA sequences to prevent amplification during PCR [12]. The suppression effect occurs when these inverted repeats anneal intramolecularly to form panhandle structures which cannot be amplified by PCR [12-16].

The DNA population formed as a result of ligation of the SO1/IR1 and SO2/IF1 DNA fragments contains several types of molecules (Figure 1 and Figure 3, lane 3): homomeric ligation products (type A), non-ligated DNA fragments (type B), and molecules representing full length initial DNA template (type C). Homomeric ligation products consist of self-ligated DNA fragments, and thus have identical ends. Heteromeric ligation products have different annealing sites for the SO1 and SO2 oligonucleotides on their 5' and 3' ends.

SO1 and SO2 oligonucleotides add suppression PCR inverted repeat elements to the ends of type A molecules. Both of these primers are long and contain the additional suppression sequences as a non-annealing overhang (Additional File 1). The overhang sequence is incorporated into template DNA ends in the early rounds of PCR. SO1 and SO2 should have a GC content of 50-70% and a T_m _of at least 65°C; whenever possible the T_m _should be greater than 70°C [15,16]. In our experience, suppression primers with an annealing temperature of 70°C give complete suppression of amplification of type A molecules. It is important to avoid using self-complementary SO1 and SO2 primer sequences which can fold back and form intramolecular hydrogen bonds, as well as primers that have complementarity to the internal mutagenic primers, particularly in their 3' ends.

The entire DNA population obtained during ligation is used for PCR with external SO1 and SO2 oligonucleotides (Figure 1 and 3). Suppression occurs when complementary sequences are present on each end of a single-stranded DNA fragment (Additional File 1). During PCR, after each denaturation step, self-complementary ends of single-stranded (ss) type A molecules form strong duplexes; thus, all ss type A molecules adopt hairpin structures (Figure 3 and Additional File 1). Replication of such DNA fragments using SO1 and SO2 oligonucleotides is suppressed (Figure 1 and Figure 3). Type B molecules have only one primer annealing site, and thus amplify linearly. Only heteromeric ligation products (type C, Figure 1 and 3) with two different adaptors at their 5' and 3' ends amplify exponentially (Figure 3, compare lines 3 and 4). These molecules are enriched during PCR and the final reaction mixture contained predominantly these DNA species (Figure 3, lane 4).

An aliquot of the PCR mixture is then directly used for the next round of mutagenesis, in this case for the sequential introduction of a 50 bp nucleotide exchange, a 50 bp insertion and a 70 bp sequence deletion, using the same strategy as described above, with the exception that internal oligonucleotides were designed for the other regions of target DNA (Table 1). An example of the resulting mutated DNA is shown in Figure 2.

**Figure 4:**
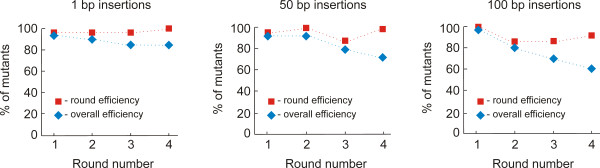
**Efficiency of MALS**. A series of sequence insertions of different lengths (indicated on the top of each graph) were introduced into the target DNA during four rounds of mutagenesis. After each round the percentage of mutants with the correct nucleotide alterations specific for particular round (round efficiency) and after all rounds (overall efficiency) were calculated. Vertical scale is percentage of mutants containing correct nucleotide alterations. Bottom scale denotes the number of rounds performed.

There is no length limitation for sequence deletions that can be introduced with MALS. The lengths of sequence insertions and nucleotide exchanges are restricted only by the available lengths of internal oligonucleotides. To estimate the efficiency of the method, we sequentially introduced a series of sequence insertions at four distinct sites into our model target DNA using the same suppression oligonucleotides SO1 and SO2 and internal mutagenic oligonucleotides listed in the Additional File 2. The mutation series included four 1 bp sequence insertions, four 50 bp insertions and four 100 bp insertions. After each round of the MALS procedure, the mutant DNA was ligated into the vector and cloned, and 20 individual plasmids with inserts were isolated and completely sequenced. In each experiment, the percentage of mutants containing correct nucleotide alterations specific for particular round (round efficiency) and number of desired mutations after all rounds (overall efficiency) were calculated (Figure 4). Under "correct DNA mutants" we list correctly assembled plasmid inserts with specific nucleotide alterations at the mutant sites. We did not take into account rare spontaneous single nucleotide exchanges introduced by DNA polymerase, because the frequencies of these errors were relatively low (0 - 20% of plasmid inserts had random nucleotide exchanges), varied from round to round, and were not reproducible. Also, the frequency of random single nucleotide exchanges will depend on the fidelity of DNA polymerase used for PCR [17,18]. Fidelity is a measure of the ability of a DNA polymerase to select a correct deoxynucleoside triphosphate from a pool of structurally similar molecules, and thus defines the probable number of random errors accumulated in the synthesized strands of DNA [18,19].

The use of high fidelity DNA polymerases in the PCR-based SDM is essential for reducing the introduction of random amplification errors in PCR products. Multiple thermostable DNA polymerases have been developed for high fidelity PCR amplification [18,19]. However, the average base substitution error rates exhibited by non-proofreading DNA polymerases (DNA polymerases having no 3'-5' exonuclease activity) are significantly higher than the error rates of proofreading enzymes [18,19]. Therefore, we suggest the use of proofreading DNA polymerases with the highest fidelity to reduce the generation of spontaneous nucleotide exchanges in the mutant DNA. Thermostable DNA polymerases having 5'-3' exonuclease activity are undesirable in the MALS procedure because protruding ends of external oligonucleotides and mismatches introduced by the mutagenic primers can be removed by 5'-3' exonuclease activity. It is also desirable to avoid using DNA polymerases which catalyze the non-template directed addition of an adenine residue to the 3' end of both strands of DNA molecules, because this will affect effective ligation of PCR fragments. Additional File 3 provides the characteristics of commercial proofreading enzymes suitable for the MALS procedure. The choice is dependent on the size of target DNA and availability of any particular enzyme.

Figure 4 shows that the efficiency of the MALS procedure was not significantly affected by the size of the sequence insertions or by the number of rounds of mutagenesis. This is because the internal oligonucleotides have predetermined mutations, thus all specific PCR fragments contain the mutations after PCR. However, undesired products accumulating during the procedure reduce the overall efficiency with increasing number of rounds. These undesired mutant products included DNA molecules containing primer dimers (the product of nonspecific annealing and primer elongation events) between the specific DNA fragments.

During PCR, formation of primer dimers can compete with formation of specific PCR product. In general, incorporation of unremoved primer dimers formed by phosphorylated internal oligonucleotides between specific PCR fragments can strongly reduce the efficiency of the mutagenesis procedure. To avoid this, efficient separation of specific PCR fragments from short primer dimers is required. We recommend the use of ion exchange chromatography or solid-phase silica-impregnated filter membranes for the separation of the specific PCR fragments from primer dimers. Commercial PCR purification systems effectively remove short ds DNA from the DNA population and are available from Promega (Wizard PCR Preps DNA purification system, which effectively removes short ds DNA up to 100 bp in length), Qiagen (QIAquick PCR Purification Kit, which completely removes ds DNA up to 50 bp in length) or other manufacturers. Alternatively, specific DNA fragments can be separated from long primer dimers (>100 bp) or excessive unspecific PCR products by gel extraction.

In our model experiments, the percentages of mutants containing the desired nucleotide alterations at all sites ranged from 60-80% after four rounds of mutagenesis had been performed. However, the efficiency of the method will depend on the specificity of oligonucleotides and their ability to generate miss-priming PCR products and, thus, will very from experiment to experiment.

## Conclusion

We report an efficient strategy for a PCR-based multiple-site SDM which employs suppression PCR for the *in vitro *selection of desired mutant DNA molecules from undesired DNA molecules. The method allows introduction of multiple complex mutations into the same target without intermediate subcloning between rounds of mutagenesis. The method is relatively simple and efficient, and it provides a number of advantages over existing commercial methods for complex mutagenesis projects.

## Methods

The following three-step protocol for one round of MALS can be easily adapted for all types of ds target DNA and used for the sequential generation of multiple mutations in the same template.

### Step 1: PCR

1 ng of genomic DNA (λ phage) was amplified separately with two pairs of oligonucleotides SO1/IR1, and SO2/IF1 (Table 1) in a 50 micro liter reactions containing 1× Pfu Ultra polymerase reaction buffer (Stratagene, La Jolla, USA), 250 μM of each dNTP, 10 picomoles of each forward and reverse oligonucleotides, and 1 unit of *Pfu Ultra *High-Fidelity DNA polymerase (Stratagene, La Jolla, USA). Cycling parameters were: 98°C for 10 sec, 60°C for 20 sec, 72°C for 5 min a total of 20 cycles. After amplification, the resulting DNA fragments were extracted from PCR mixtures using Wizard PCR Preps DNA Purification System (Promega, Madison, USA). This purification procedure is required for separation of specific DNA fragments from primer dimers if they are formed during PCR.

### Step 2: Ligation

Equimolar amounts of DNA fragments obtained in Step 1 were ligated to each other in a 20 μl reaction containing 50 ng of SO1/IR1 DNA fragment, 200 ng of SO2/IF1 DNA fragment, 1× T4 DNA ligase reaction buffer and 1 μl of high concentrated (30,000 Weiss units/ml) T4 DNA ligase (#M0202 M, New England Biolabs, Ipswich, USA) for 15-30 min at room temperature. An aliquot of this ligation mixture was then diluted 100 fold for subsequent suppression PCR.

### Step 3: Suppression PCR

Ligated DNA fragments from Step 2 were amplified with SO1 and SO2 suppression oligonucleotides in a 50 μl reaction containing 1 μl of diluted ligation mixture, 1× Pfu Ultra polymerase reaction buffer (Stratagene, La Jolla, USA), 250 μM of each of the dNTP, 10 picomoles of each SO1 and SO2 oligonucleotides, and 1 unit of *Pfu Ultra *High-Fidelity DNA polymerase (Stratagene, La Jolla, USA). PCR reactions were done under conditions as follows: 98°C for 10 sec, 68°C for 6 min for a total of 20 cycles. An aliquot of final PCR mixture was diluted 100 fold and mutagenesis procedure was sequentially repeated with other mutagenic oligonucleotides listed in Table 1.

After completion the last round of mutagenesis, the suppression sequences were removed from DNA fragments with *Hind*III and *Nhe*I restriction endonucleases. The resulting DNA fragments were ligated with *Hind*III/*Nhe*I linearized plasmid vector and subcloned. Vector inserts were then sequenced in both directions using BigDye Terminator v3.1 chemistry and a 3130xl Genetic Analyzer (Applied Biosystems, CA, USA) to verify the presence of desired mutations.

## Abbreviations

SDM: Site-Directed Mutagenesis.

## Competing interests

The authors declare that they have no competing interests.

## Authors' contributions

A.F. conceived the method, generated the experimental data, and wrote the first draft of the manuscript. D.D. participated in the evaluation of the data, revised the manuscript, and gave final approval for the version to be published.

## Supplementary Material

Additional file 1 **Structure of suppression oligonucleotides and the effect of PCR suppression**. Supplemental figure showing detailed structure of SO1 and SO2 suppression oligonucleotides and principle of PCR suppression. Click here for file

Additional file 2 **Mutagenic oligonucleotides used in this study**. Supplemental Table S1 listing oligonucleotides used in this study. Click here for file

Additional file 3 **Characteristics of modern high fidelity DNA polymerases**. Supplemental Table S2 describing characteristics of commercial high fidelity DNA polymerases. Click here for file
