# Supplementary material for: MALS: an efficient strategy for multiple site-directed mutagenesis employing a combination of DNA amplification, ligation and suppression PCR
Source: BMC Biotechnol. 2009 Sep 24;9:83. doi: 10.1186/1472-6750-9-83 (PMC2759926; doi:10.1186/1472-6750-9-83)
Supplement: Additional file 1 — Structure of suppression oligonucleotides and the effect of PCR suppression. Supplemental figure showing detailed structure of SO1 and SO2 suppression oligonucleotides and principle of PCR suppression. [file 1472-6750-9-83-S1.pdf]

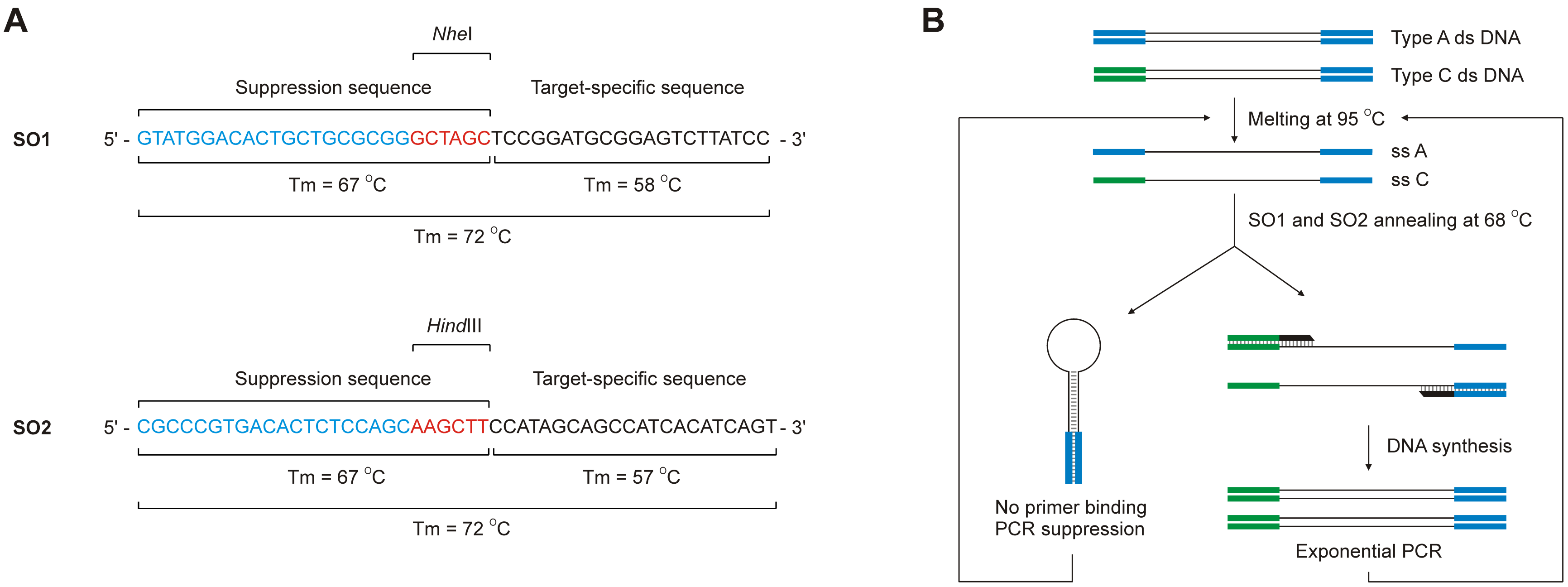

Additional File 1. Structure of suppression oligonucleotides and the effect of PCR suppression. (**A**) Nucleotide sequences of SO1 and SO2 suppression oligonucleotides consisting of suppression sequences (5' stretches of 26 nucleotides) and target-specific sequences. *Nhe*I and *Hind*III restriction recognition sites are in red. The 5' ends of both oligonucleotides are unphosphorylated. Melting temperatures (T_m) are indicated separately for suppression parts, target-specific parts, and for whole oligonucleotides. (**B**) Scheme of suppression PCR. Suppression occurs when complementary sequences are present on each end of a single-stranded (ss) DNA fragment. During each primer annealing step, the hybridization kinetics of the type A ss DNA molecules favor the intramolecular formation of a secondary structure that prevents primer annealing. During PCR, replication of type A molecules is efficiently suppressed, and amplification of type C DNA molecules with different adaptors at both ends proceeds normally.
