# Supplementary material for: MALS: an efficient strategy for multiple site-directed mutagenesis employing a combination of DNA amplification, ligation and suppression PCR
Source: BMC Biotechnol. 2009 Sep 24;9:83. doi: 10.1186/1472-6750-9-83 (PMC2759926; doi:10.1186/1472-6750-9-83)
Supplement: Additional file 2 — Mutagenic oligonucleotides used in this study. Supplemental Table S1 listing oligonucleotides used in this study. [file 1472-6750-9-83-S2.pdf]

Supplemental Table 1. Mutagenic oligonucleotides used in this study.

Name	Nucleotide sequence 5'-3'	Orientation	Mutation introduced
Internal mutagenic oligonucleotides for introducing of series of 1 bp insertions (mutagenic nucleotides are underlined)			
IS1 F1	GTCCAGACCCAACCAAACCAATCG	Forward	Each pair of oligonucleotides introduces a 1 bp insertion (underlined) at one of the four distinct sites
IS1 R1	AGGATTTGCTGTACTGCGTGAATAGC	Reverse	
IS1 F2	GTCAGGTTGAATGGCATGGTCGCT	Forward	
IS1 R2	CGAGAGACGTCACCTAAGCAGG	Reverse	
IS1 F3	GCGTGTAGGCGAATTTGCGGAG	Forward	
IS1 R3	CATCCTGCTGGTTGACTGGCCTA	Reverse	
IS1 F4	GCTCCGGTGGCAGGACGTCAGCA	Forward	
IS1 R4	AAACTAACGACATTTATCATGCAGCC	Reverse	
Internal mutagenic oligonucleotides for introducing of series of 50 bp insertions			
IS50 F1	ATTTGCAATCCCAATGATTGAACTATCCAGACCCAACCAAACCAATCG	Forward	Each pair of oligonucleotides introduces a 50 bp insertion (underlined) at one of the four distinct sites
IS50 R1	ATCTCGGAGCTCGCTCGCATAAGCAAGGATTTGCTGTACTGCGTGAATAGC	Reverse	
IS50 F2	GTTCCGAGATAAGTTTACGTCACCGTCAGGTTGAATGGCATGGTCGCT	Forward	
IS50 R2	GTTTGTCCAGACTAAAAATTCAGATCGAGAGACGTCACCTAAGCAGG	Reverse	
IS50 F3	TTAGCTTTTATCGGCCAATTCCATGCGTGTAGGCGAATTTGCGGAG	Forward	
IS50 R3	ACAGCTAATAATGTTGTATATTGGTCATCCTGCTGGTTGACTGGCCTA	Reverse	
IS50 F4	TCCTATTCGTCGACCACACCTGAACCTCCGGTGGCAGGACGTCAGCA	Forward	
IS50 R4	TAATCTGATTTCGGGAACGCCACTTAAACTAACGACATTTATCATGCAGCC	Reverse	
Internal mutagenic oligonucleotides for introducing of series of 100 bp insertions			
IS100 F1	ACTCCGGTATCCCATGCGCGAAGAGTGCGTTCTTATTGACCTGGGCCTAATCCAGACCCAACCAAACCAATCG	Forward	Each pair of oligonucleotides introduces a 100 bp insertion (underlined) at one of the four distinct sites
IS100 R1	CTCGACAAAGTCTACGAAGTATGTCTCTTGATATGCAGACGAGCAGGTACAGGATTTGCTGTACTGCGTGAATAGC	Reverse	
IS100 F2	CGCTGGTTATCGGAACGTCCGTTCGTTTCGACTTTTCCGGAGTCTTTCACTCAGGTTGAATGGCATGGTCGCT	Forward	
IS100 R2	TCAGTGATAGCCAGAGGTATTCTGTTTTTGTTTACCTGGTGACCGTGAGTCGAGAGACGTCACCTAAGCAGG	Reverse	
IS100 F3	GACAATTCTCAGTTGGCACCTCTATAATACCAGGCCACGATACATGTTGACGTGTAGGCGAATTTGCGGAG	Forward	
IS100 R3	CTTTTAGCACGGTATAGATCCGAATTGTGTAGCTCGTCGCGAAGTGAGTCCATCCTGCTGGTTGACTGGCCTA	Reverse	
IS100 F4	ATGGCGAATGATGGGCGTTGTTGTCAGAAGCAAGTTGTGGGACTAGGTATCTCCGGTGGCAGGACGTCAGCA	Forward	
IS100 R4	TGGTATTCATGACGGCTCTGGCCCGATTTCTTGGCCCACTAGCGCAATACAAACTAACGACATTTATCATGCAGCC	Reverse	
