# Supplementary material for: MALS: an efficient strategy for multiple site-directed mutagenesis employing a combination of DNA amplification, ligation and suppression PCR
Source: BMC Biotechnol. 2009 Sep 24;9:83. doi: 10.1186/1472-6750-9-83 (PMC2759926; doi:10.1186/1472-6750-9-83)
Supplement: Additional file 3 — Characteristics of modern high fidelity DNA polymerases. Supplemental Table S2 describing characteristics of commercial high fidelity DNA polymerases. [file 1472-6750-9-83-S3.pdf]

Supplemental Table 2. Characteristics of modern high fidelity DNA polymerases.

Product name	DNA polymerase derivative	Manufacturer	Exonuclease activity		Recommended maximal target length (genomic/vector, kb)	Elongation rate (bases/second)	Error rate* ($\times 10^{-6} \pm$ S.D.)	Accuracy* (error rate^{-1} in bases)	Percentage of clones with mutations (10^6-fold amplification)**			Reference
			3'-5'	5'-3'					1-kb amplicon	5-kb amplicon	10-kb amplicon	
Pfu	*Pyrococcus furiosus*	Stratagene	yes	no	4/10	8-17	1.3 ±0.2	770000	2.6	13	26	S1-S3
PfuTurbo	*Pyrococcus furiosus*	Stratagene	yes	no	15/19	25	1.3 ±0.2	770000	2.6	13	26	S1-S3
PfuUltra	*Pyrococcus furiosus*	Startagene	yes	no	6/17	25	0.4 ±0.04	2500000	0.8	4	8	S1-S3, S5
Tgo	*Thermococcus gorgonarius*	Roche	yes	no	3/<5	-	2.2 ±0.1	450000	4.4	n.r.	n.r.	S1, S3
DeepVent$_R$	*Pyrococcus* sp. GB-D	NEB	yes	no	-	23	2.7 ±0.2	370000	5.4	n.r.	n.r.	S1, S2, S4
Vent$_R$	*Thermococcus litoralis*	NEB	yes	no	-	16	2.8 ±0.9	360000	5.6	n.r.	n.r.	S1, S2, S4
Platinum Pfx	*Thermococcus kodakaraensis* KOD 1	Invitrogene	yes	no	12/20	67	3.5 ±1.0	290000	7	35	70	S1, S3
Phusion High Fidelity	*Pyrococcus sp*. GB-D	Finnzymes	yes	no	7.5/20	70	0.4 ±0.04	n.d.	1.32	n.d.	n.d.	S5
KOD Hi Fi	*Thermococcus kodakaraensis* KOD 1	Toyobo, Novagen	yes	no	2/6	106-138	3.5 ±1.0	290000	7	35	n.d.	S1, S6

* - error rate (mutation frequency per base pair per duplication) and accuracy of DNA polymerases were measured by authors using a PCR forward mutation assay which measures the frequency of mutations introduced into the *lacI* target gene during PCR amplification (S1, S2).
** - based on the error rate, the percentage of mutated PCR products were calculated for a 1, 5 or 10 kb target DNA sequence for 20 effective cycles.

Abbreviations: n.r., DNA polymerase is not recommended for that target DNA size; n.d., not determined.

References

S1. Hogrefe HH and Borns M: **High fidelity PCR enzymes.** In: *PCR Primer: A Laboratory Manual*. 2nd edition. Edited by Dieffenbach CW and Dveksler GS. New York: Cold Spring Harbor Laboratory Press; 2003:21-34.

S2. Cline J, Braman JC, Hogrefe HH: **PCR fidelity of pfu DNA polymerase and other thermostable DNA polymerases**. *Nucleic Acids Res* 1996, **24**(18):3546-3551.

S3. Arezi B, Xing W, Sorge JA, Hogrefe HH: **Amplification efficiency of thermostable DNA polymerases**. *Anal Biochem* 2003, **321**(2):226-235.

S4. Kong H, Kucera RB, Jack WE: **Characterization of a DNA polymerase from the hyperthermophile archaea Thermococcus litoralis. Vent DNA polymerase, steady state kinetics, thermal stability, processivity, strand displacement, and exonuclease activities**. *J Biol Chem* 1993, **268**(3):1965-1975.

S5. Li M, Diehl F, Dressman D, Vogelstein B, Kinzler KW: **BEAMing up for detection and quantification of rare sequence variants**. *Nat Methods* 2006, **3**(2):95-97.

S6. Takagi M, Nishioka M, Kakihara H, Kitabayashi M, Inoue H, Kawakami B, Oka M, Imanaka T: **Characterization of DNA polymerase from Pyrococcus sp. strain KOD1 and its application to PCR**. *Appl Environ Microbiol* 1997, **63**(11):4504-4510.
